# Determining the sex-specific distributions of average daily alcohol consumption using cluster analysis: is there a separate distribution for people with alcohol dependence?

**DOI:** 10.1186/s12963-021-00261-4

**Published:** 2021-06-07

**Authors:** Huan Jiang, Shannon Lange, Alexander Tran, Sameer Imtiaz, Jürgen Rehm

**Affiliations:** 1 Institute for Mental Health Policy Research, Centre for Addiction and Mental Health (CAMH), 33 Ursula Franklin Street, Toronto, Ontario M5S 2S1 Canada; 2 Dalla Lana School of Public Health, University of Toronto, 6th Floor, 155 College Street, Toronto, Ontario M5T 3M7 Canada; 3 Institute of Clinical Psychology and Psychotherapy & Center for Clinical Epidemiology and Longitudinal Studies, Technische Universität Dresden, Chemnitzer Str. 46, D-01187 Dresden, Germany; 4 Campbell Family Mental Health Research Institute, CAMH, 250 College Street, Toronto, Ontario M5T 1R8 Canada; 5 Department of Psychiatry, University of Toronto, 8th Floor, 250 College Street, Toronto, Ontario M5T 1R8 Canada; 6 Institute of Medical Science, University of Toronto, 1 King’s College Circle, Toronto, Ontario M5S 1A8 Canada; 7 Department of International Health Projects, Institute for Leadership and Health Management, I.M. Sechenov First Moscow State Medical University, Trubetskaya str., 8, b. 2, Moscow, Russian Federation 119992; 8 Center for Interdisciplinary Addiction Research (ZIS), Department of Psychiatry and Psychotherapy, University Medical Center Hamburg-Eppendorf (UKE), Martinistraße 52, 20246 Hamburg, Germany

**Keywords:** Alcohol consumption, Machine learning, survey, Gaussian Mixture Models, Clustering, Alcohol use disorders, Treatment utilization

## Abstract

**Background:**

It remains unclear whether alcohol use disorders (AUDs) can be characterized by specific levels of average daily alcohol consumption. The aim of the current study was to model the distributions of average daily alcohol consumption among those who consume alcohol and those with alcohol dependence, the most severe AUD, using various clustering techniques.

**Methods:**

Data from Wave 1 and Wave 2 of the National Epidemiologic Survey on Alcohol and Related Conditions were used in the current analyses. Clustering algorithms were applied in order to group a set of data points that represent the average daily amount of alcohol consumed. Gaussian Mixture Models (GMMs) were then used to estimate the likelihood of a data point belonging to one of the mixture distributions. Individuals were assigned to the clusters which had the highest posterior probabilities from the GMMs, and their treatment utilization rate was examined for each of the clusters.

**Results:**

Modeling alcohol consumption via clustering techniques was feasible. The clusters identified did not point to alcohol dependence as a separate cluster characterized by a higher level of alcohol consumption. Among both females and males with alcohol dependence, daily alcohol consumption was relatively low.

**Conclusions:**

Overall, we found little evidence for clusters of people with the same drinking distribution, which could be characterized as clinically relevant for people with alcohol use disorders as currently defined.

## Introduction

Alcohol use is a major risk factor for burden of disease [1], and alcohol use disorders (AUDs) comprise a substantial part of the alcohol-attributable disease burden globally [2]. While it is obvious that an individual who completely abstains from consuming alcohol would not be diagnosed with an AUD—i.e., such use is, by definition, a necessary and sufficient cause (see [3, 4] for further discussion), there is a great deal of debate in the literature regarding whether or not these conditions can be characterized by specific levels of daily alcohol consumption. In both the Diagnostic and Statistical Manual of Mental Disorders, 5th edition (DSM-5) and the International Classification of Diseases, 11th revision (ICD-11) [5, 6], AUDs are defined without any reference to the level of drinking, but rather, are based on a non-specific set of behavioral, social, psychological, and physiological criteria [7, 8]—e.g., continuing to drink even though it is causing trouble with friends/family, and giving up or cutting back on activities that were once important or interesting in order to drink. While these definitions have been criticized [9, 10], level of drinking, as an alternative key criterion, may pose other concerns (see [10, 11] for further discussion), such as:

- What would be the thresholds for minimal level of alcohol use, and for what duration would one need to consume the respective level of alcohol in order for a diagnosis to be justified?
- Would heavy use over time be sufficient for a diagnosis on its own, or would additional criteria, such as withdrawal or tolerance, be necessary, and should brain function also be considered [8]?

As for thresholds, a similar approach could be used as the one used for hypertension [12]. Even though blood pressure is a continuous variable, and the higher the blood pressure, the higher the chance for various disease categories [13], expert committees do not seem to have a problem agreeing on the blood pressure levels for which an intervention is required (in other words, to determine the threshold, when the blood pressure level is high enough to be considered a disease, e.g., see [14]). However, given the modern techniques available for analyzing distributions, more empirical-based methods may differentiate seemingly continuous distributions with only one maximum [15, 16]. When it comes to alcohol use, these techniques assume that the supposed unimodal distribution is a mixture of different groups of individuals with different distributions of alcohol use, which can be detected via machine learning techniques. For instance, clustering, an unsupervised learning technique, is often used to find clusters of points that appear close together [17, 18].

In the current study, we have taken this approach and have hypothesized that (1) the (sex-specific) distributions of average daily alcohol consumption among those who consume alcohol can be described as mixture models and thus, are best represented by more than one distribution; (2) individuals with alcohol dependence, the most severe AUD, can be characterized by one of these distributions of average daily alcohol consumption; and (3) that treatment utilization will be associated with distributions of daily alcohol consumption among individuals with alcohol dependence. All analyses were sex-specific given the different body composition and the neurobiological processes of alcohol use and AUDs of males and females [19], as well as the sex differences in the quantity and frequency of alcohol use [20] and treatment utilization rates among individuals with an AUD [21].

## Methods

### Data source

The current analysis is based on data from Wave 1 and Wave 2 of the National Epidemiologic Survey on Alcohol and Related Conditions (NESARC), designed and sponsored by the National Institute on Alcohol Abuse and Alcoholism, conducted in 2001–2002 and 2004–2005, respectively. The NESARC sample represents the civilian, noninstitutionalized adult population of the USA [22]. The surveys were conducted using face-to-face, computer-assisted, and in-home interviews. One randomly selected adult (aged 18 years or older) from each sampled household was invited to participate. The overall response rate was 81.0% for Wave 1, for a total sample size of 43,093. Among those, 34,653 (80.4%) were followed-up in Wave 2 (8840 participants were lost to follow-up).

The NESARC samples were weighted to adjust for probabilities of selection biases and nonresponse. Calibration was applied to match the target population based on the 2000 census. Details regarding the sampling, weighting, and calibration have been described elsewhere [23, 24].

### Measures

#### Daily alcohol consumption

The NESARC contains detailed questions about the drink types, frequency of drinking, and quantity and size of drinks consumed during the past 12 months. The amount of pure alcohol in each drink was calculated using an ethanol conversion factor, which accounts for the proportion of pure alcohol in the different types of drinks [23, 24]. The average daily volume of pure alcohol consumption in grams during the past 12 months (referred to as daily alcohol consumption herein) was then calculated by dividing the total alcohol consumption across all drink types by 365. This variable did not only include quantity and frequency of consumption, but also adjusted for heavy drinking occasions (see Appendix 3 for details).

#### Alcohol dependence

Alcohol dependence in the past 12 months was assessed using the Alcohol Use Disorders and Associated Disabilities Interview Schedule-IV (AUDADIS-IV), based on the criteria of the fourth edition of the DSM (DSM-IV) [25, 26].

#### Treatment utilization

The NESARC defines broadly alcohol treatment utilization as “seeking help for alcohol-related problems” from at least one of the following: alcoholics/narcotics/cocaine anonymous, or 12-step meeting; family services or other social service agency; alcohol/drug detoxification ward/clinic; inpatient ward of psychiatric/general hospital or community mental; outpatient clinic, including outreach and day/partial patient programs; alcohol/drug rehabilitation program; emergency room because of drinking; halfway house/therapeutic community; crisis center because of drinking; employee assistance program; clergyman, priest, or rabbi; private physician, psychiatrist, psychologist, social worker, or any other professional; and any other agency or professional. Accordingly, for Wave 1, treatment utilization was considered as the endorsement of any of the above within the past 12 months. For Wave 2, alcohol treatment utilization was ascertained using the following question: “Have you gone anywhere or seen anyone to get help because of drinking since last interview?”

### Statistical analysis

As an exploratory analysis, following traditional fitting of distributions for alcohol use [27, 28], we evaluated the fit of the Log-Normal, Gamma, and Weibull distributions to determine if the distribution of daily alcohol consumption could be appropriately described as unimodal, using the Wave 1 survey. The three fittings were examined using the Kolmogorov-Smirnov test, and the null hypothesis was rejected for all three, which suggested the possibility of a multi-modal distribution. Given the skewness of the data, a log transformation was applied to the daily alcohol consumption variable, and the distribution was modelled and fitted using the following steps:

1. Density plots

Density plots of daily alcohol consumption were produced, and the resulting graphs were used to visually identify the possible number of modes.

2. Clustering

Clustering algorithms were used to group a set of data points into clusters, so that data points in the same cluster were more similar to each other than data points in other clusters. The desired number of clusters was decided using the NBClust package, which simultaneously varies the number of clusters, the clustering method and the indices to find the optimal number of clusters for the data points [29]. When the indices failed to suggest the best clustering scheme, K-means was used to select the number of desired clusters [30].

3. Gaussian Mixture Model

Given the number of clusters, Gaussian Mixture Models (GMMs) [18] were used to estimate the likelihood that a given point belonged to one of a mixture of Gaussian distributions. The mixture distribution can be represented by writing the distribution function (*F*) as a sum:

$$ F(x)=\sum \limits_{i=1}^k{w}_i{P}_i(x) $$

where *k* is the number of clusters, and *x* represents the data points and weights ${\sum}_{i=1}^n{w}_i=1$.

*P(x)* was assumed to follow Gaussian distributions. For each distribution, there are two parameters to describe the shape of the clusters: the mean and the standard deviation. The parameters were estimated via the Expectation-Maximization algorithm. There are two key advantages to using GMMs. Firstly, GMMs are more flexible in terms of cluster covariance. Secondly, since GMMs use probabilities, each data point can have multiple clusters. Therefore, if a data point is located in the middle of two overlapping distributions, its class can be defined by a mixed membership. The Bayesian Information Criterion was used to assess model fit. Sex-specific models were fit and visualized, as well as separate models for those with alcohol dependence.

Lastly, to determine the stability of the identified clusters over time, Wave 2 data were used to test if the respective mixture of distributions described using GMMs, were consistent with the distributions identified for Wave 1. In addition, a subgroup analysis using the same statistical approach, as described above, was performed on Wave 1 and Wave 2 data combined to investigate the distributions among those individuals with alcohol dependence in both waves. Individuals were assigned to the cluster for which they had the highest posterior probability from the GMMs. Treatment utilization rate were then calculated for each of the clusters based on lifetime treatment (seeking treatment prior to Wave 2) or any recent treatment (seeking treatment within the 12 months prior to Wave 1 and Wave 2).

## Results

**Fig. 1:**
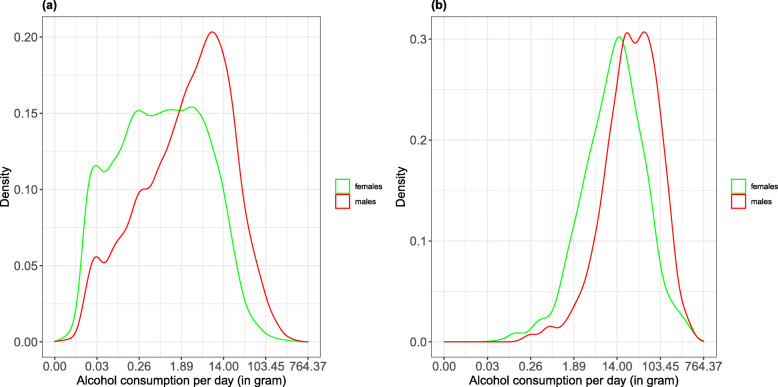
Density plots of daily alcohol consumption (log-transformed) from Wave 1 for (**a**) all individuals and (**b**) those individuals with alcohol dependence, by sex

There were more females than males in both Wave 1 and Wave 2 (52.1% vs. 47.9%; Table 1). About 4% of participants were diagnosed with alcohol dependence in both Waves (3.8% in Wave 1, and 4.4% in Wave 2. Among them, approximately two out of three were male and one out of three was female, in both waves. The density plots suggested that the daily alcohol consumption data might be grouped into two or more different clusters, which were different for males and females, and for those individuals with and without alcohol dependence (Fig. 1). As expected, male drinkers, as well as males with alcohol dependence had higher amounts of daily alcohol consumption, on average, compared to female drinkers and females with alcohol dependence, respectively.

**Table 1:** Weighted demographic information for Wave 1 and Wave 2

	Wave 1	Wave 2
	*N* (%)^a^	*N* (%)
Gender		
Females	22,443 (52.1)	18,048 (52.1)
Males	20,650 (47.9)	16,605 (47.9)
Age		
≤ 20	1698 (3.9)	0 (0.0)
20–40	16,358 (38.0)	12,144 (35.1)
41–70	20,000 (46.4)	17,592 (50.8)
71+	5038 (11.7)	4913 (14.2)
Alcohol dependence, past 12 months	1640 (3.8)	1521 (4.4)
Females	521 (31.8)	478 (31.4)
Males	1119 (68.2)	1043 (68.6)
Alcohol treatment utilization^b^	246 (0.6)	473 (1.4)

^a^*N* is adjusted by the sample weight

^b^Alcohol treatment utilization is broadly defined as “seeking help for alcohol-related problems” from at least one of 13 sources (e.g., health care facilities, alcohol-related programs, family or social services, employee assistance program or religious services)

**Fig 2:**
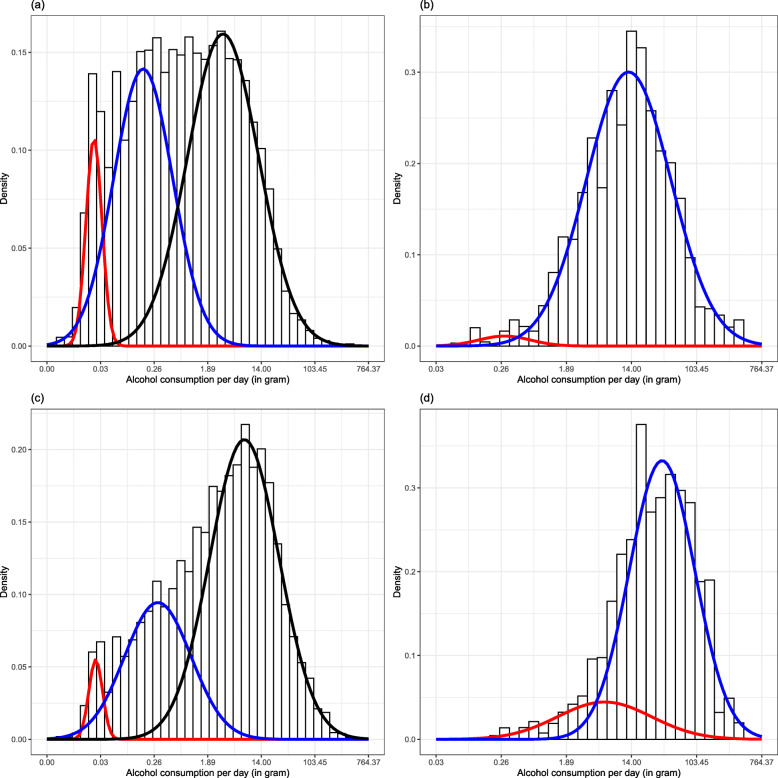
Gaussian Mixture Models for (**a**) females, (**b**) females with alcohol dependence, (**c**) males, and (**d**) males with alcohol dependence, using Wave 1 data

The log-transformed daily alcohol consumption of all individuals in Wave 1 was best described by a three-component GMM for both females and males (Fig. 2). The means of the three Gaussian clusters were 0.03, 0.17 and 3.34 g per day for females, and 0.03, 0.35, and 7.48 g per day for males. In contrast, a two-component GMM best described the log-transformed daily alcohol consumption for both females and males with alcohol dependence. The means of the two Gaussian clusters were 0.28 and 12.82 g per day for females with alcohol dependence, with only 2.2% of females being included in the first cluster and 97.8% included in the second cluster, and 6.12 and 35.80 g per day for males with alcohol dependence, with 16.1% of males being included in the first cluster and 83.9% included in the second cluster.

Wave 2 yielded similar results to Wave 1 in that the log-transformed daily alcohol consumption of both females and males was best described by three-component GMMs (with means of 0.03, 0.24, and 4.86 g per day, and 0.03, 0.34, and 7.48 g per day, respectively, for the three Gaussian distributions), and daily alcohol consumption of males with alcohol dependence being best described by a two-component GMM (with means of 6.12 and 35.80 g per day; see Table 2 for details and Appendix Table 4 for corresponding results in drinks per day). However, in Wave 2, daily alcohol consumption of females with alcohol dependence was best described by a three-component GMM, with the means corresponding to 3.36, 10.47, and 32.07 g per day.

**Table 2:** Summary of the sex-specific Gaussian Mixture Models for drinkers overall and for individuals with alcohol dependence using Wave 2 data

	Females	Males	Females with alcohol dependence	Males with alcohol dependence
*N*	10,970	11,897	207	364
First component				
Average daily consumption (g)	0.03	0.03	3.36	6.12
Weight	0.06	0.03	0.60	0.16
Second component				
Average daily consumption (g)	0.24	0.34	10.47	35.80
Weight	0.52	0.31	0.29	0.84
Third component				
Average daily consumption (g)	4.86	7.48	32.07	n/a
Weight	0.42	0.66	0.12	n/a

*g* grams, *n/a* not applicable, as model only yielded two distributions

Overall, the clusters identified did not point to alcohol dependence as a separate cluster characterized by a higher level of alcohol consumption. Among both females and males with alcohol dependence, daily alcohol consumption was relatively low, with the highest mean of any cluster being around 32 and 36 g per day, respectively—i.e., less than three US standard drinks.

**Fig. 3:**
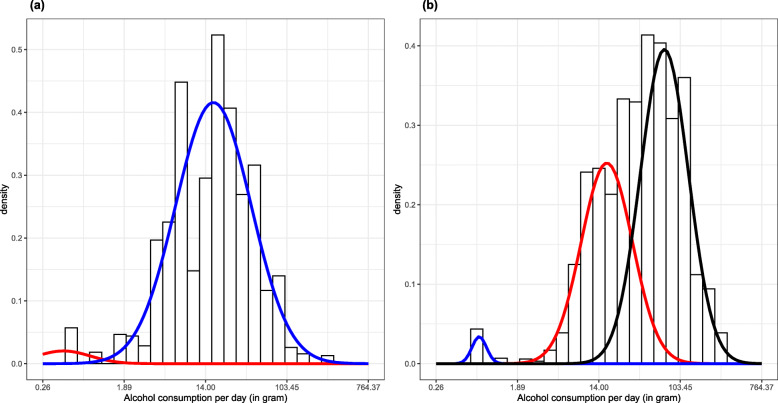
Gaussian Mixture Models for (**a**) females and (**b**) males with alcohol dependence in both Wave 1 and Wave 2

Lastly, data from Wave 1 and Wave 2 were combined to investigate the sex-specific distributions of daily alcohol consumption for those with alcohol dependence in both waves. As shown in Fig. 3, average daily alcohol consumption of females with alcohol dependence was best described by a two-component GMM, while average daily alcohol consumption of males with alcohol dependence was best described by a three-component GMM. For females with alcohol dependence in both waves, 3.3% of them belonged to a cluster with a mean of 0.42 g per day, while 96.7% of them belonged to a cluster with mean of 16.97 grams per day, de facto indicating the presence of a cluster comprised of the overwhelming majority of females with alcohol dependence with a mean drinking level of less than two drinks, which is not even considered heavy drinking (e.g., [31])

For males with alcohol dependence in both waves, the means of the three Gaussian clusters were 0.74, 17.01, and 69.99 g per day, with 1.5%, 39.9%, and 58.6% of males belonging to the respective clusters. It is only in this analyses that we identified one cluster that had a similar level of daily alcohol consumption, on average, as “typical” treatment populations (i.e., a cluster of males with alcohol dependence in both waves consumed a mean of about 70 grams of alcohol on average per day). Table 3 shows the characteristics of individuals in each of the clusters, including the percentage who had utilized treatment, their age at Wave 1, and daily alcohol consumption in grams. Around 36.0% of males in the third cluster utilized alcohol-related treatment, compared with 21.5% of males in the second cluster. In other words, we identified a cluster of males who could be described as those with alcohol dependence requiring treatment. Whereas, the first, very small cluster among the group of males with alcohol dependence in both waves, likely consists of people who recently completed treatment, and were still abstinent. Appendix Table 5 shows the further details of those three clusters.

**Table 3:** Cluster descriptions for those with alcohol dependence in both Wave 1 and Wave 2

	*N*	Treatment utilization, lifetime (%)	Treatment utilization, past 12 months at Wave 1 and/or between Wave 1 and Wave 2 (%)	Age (years) at Wave 1—mean (SD)	Average daily alcohol consumption (g)—mean (SD)
Females					
Cluster 1	4	0.0	0.0	20.98 (5.84)	0.47 (0.25)
Cluster 2	126	31.4	21.5	34.94 (11.34)	25.67 (27.99)
Males					
Cluster 1	5	86.3	86.3	31.24 (3.10)	0.75 (0.16)
Cluster 2	131	35.7	21.5	31.31 (11.12)	17.09 (7.50)
Cluster 3	201	55.9	36.0	32.98(12.03)	84.43 (49.46)

*g* grams, *SD* standard deviation

## Discussions

A procedure for deriving different distributions of daily alcohol consumption based on a statistical clustering methodology was presented and explored. This procedure allowed for the quantitative comparison of the distributions between surveys conducted on the same individuals at different time points. Overall, we found little evidence for clusters of people with the same drinking distribution, which could be characterized as clinically relevant for people with AUDs, as currently defined. Before we discuss the results further, we would like to discuss a few potential limitations of the approach taken.

To begin, the selection of number of clusters was done through calculating multiple indices and the clustering scheme with the most agreement was adopted. When the sample size was small, the indices were less likely to agree with each other. In the case of algorithm failure and limited computing capacity, the K-means method was used to choose an appropriate number of clusters. This method assumes that all clusters are equally sized and have the same variance. When analyzing average daily alcohol consumption for females and males in the overall sample, the sample sizes were large and the variances of all clusters were well balanced. However, the clusters for females and males with alcohol dependence in both Wave 1 and Wave 2, given the relatively small sample sizes, may not have been stable enough to be generalizable.

GMMs are primarily used in modeling populations with multiple distributions and have gained prominence within the model-based clustering framework. Using GMMs, we are able to identify occasional drinkers, light drinkers, and heavy drinkers. Given the skewed distribution of daily alcohol consumption, a log transformation was used to make the data conform to normality, which was a requirement for the methodology used. However, it may be more difficult to interpret the findings that are based on transformed data with respect to the hypotheses of interest.

Lastly, the study was based on a survey, where the assessment of alcohol consumption was based on self-reported data, which are known to underestimate true consumption levels [32, 33], either due to restricted sampling frame or due to reporting biases [34]. It is unclear how these biases impacted the current findings, but it is very likely that heavy alcohol consumers were not part of the sample [35], which is exemplified by the fact that the homeless, institutionalized, or members of the army living on base were not included. Finally, as with all surveys, the NESARC had high, but less than perfect participation rates, and some loss to follow-up, which could impact the results.

Of all the clusters identified, only the cluster with the highest average daily alcohol consumption among males with alcohol dependence in both survey waves showed drinking levels likely seen in treatment populations in North America (e.g., see [36, 37]), with European samples often showing higher levels [38, 39]. In summary, we did not identify a cluster which could be characterized as AUD among the general drinking population or individuals with alcohol dependence at one time point only, but may have identified a cluster among males with alcohol dependence in both waves. In other words, we failed to corroborate hypothesis 2 above, which postulated that individuals with alcohol dependence can be characterized by one of these distributions of average daily alcohol consumption.

There are a few reasons for this result, which is different from other diseases and underlying biomarkers—for example, blood glucose levels and diabetes (e.g., see [40]). First, the current criteria for AUDs are very inclusive [7], and likely do not represent need for treatment intervention, and may not grasp what has been traditionally understood as “addiction,” often defined by treatment populations [4]. Consider the following: in a treatment-based sample, lifetime alcohol dependence was indeed stable, with approximately 90.5% for females and 94.7% for males, whereas in a community-based sample the stability of lifetime alcohol dependence was only 27.5% and 64.7% for females and males, respectively [41]. The most important characteristic that determined diagnostic stability was severity. Thus, a diagnosis which by definition should not change, was stable in severe, treatment-seeking cases, but not in general population cases of alcohol dependence, and alcohol dependence is already the more severe AUD in the DSM-IV. In other words, measurement of AUD in the general population picks up a lot of very mild cases that are not necessarily indicative of alcohol addiction or problem-drinking. Cases in which individuals are consuming alcohol more regularly appear relatively mild given that they are often forgotten by respondents when asked the same questions later.

However, the question “why can we not pick up stable groups of heavy or very heavy drinkers [42] with the current methodology” remains. We can offer three potential reasons here: first, that many people with severe alcohol dependence fall out of the sampling frame of general population surveys, as they are not living in households sampled, but are homeless or institutionalized [35]. Second, they may not participate even if they had been part of the sampling frame [43]. Or, third, people with an AUD do not distinctively differ in their level of drinking as a subgroup of the general population, which would be in line of theories such as the one brought forward by Ledermann [15, 16, 44]. It would be important to clarify these questions, even though it is not easy given the current status of general population surveys [34].

## Conclusions

The clustering procedure proved feasible for modeling average daily alcohol consumption based on survey data and allowed for the quantitative differentiation of the distributions among the study populations. We concluded that AUDs as currently defined could not be described by a group of people based on average daily alcohol consumption. This conclusion could reflect a problem with the underlying definitions which may be too unspecific and wide [4]. However, it may also reflect the fact that people with AUD do not represent a distinct category with respect to alcohol use, but one group within a continuum of use, as described by Skog [16] or Lederman [15]. The answer to this question not only would increase current knowledge, but also has practical implications for conceptualizing disease and clinical care.

## Data Availability

The R Code used to analyze and compute variables for the current study are available from the corresponding author upon reasonable request. The datasets supporting the conclusions of this article are available in the National Institute on Alcohol Abuse and Alcoholism repository [http://niaaa.census.gov/data.html].

## Appendix 1

**Table 4:** Results of the sex-specific Gaussian Mixture Models for drinkers overall and for individuals with alcohol dependence using Wave 2 data: log-transformed alcohol consumption in drinks as the dependent variable

	Females	Males	Females with alcohol dependence	Males with alcohol dependence
First component				
Mean	− 6.30	− 6.15	− 1.43	− 0.83
Exp (mean)*	0.00	0.00	0.24	0.44
SD	0.25	0.26	1.40	1.44
Weight	0.06	0.03	0.60	0.16
Second component				
Mean	− 4.05	− 3.69	− 0.29	0.94
Exp (mean)*	0.02	0.02	0.75	2.56
SD	1.26	1.22	0.50	1.01
Weight	0.52	0.31	0.29	0.84
Third component				
Mean	− 1.06	− 0.63	0.83	n/a
Exp (mean)*	0.35	0.53	2.29	n/a
SD	1.10	1.23	0.29	n/a
Weight	0.42	0.66	0.12	n/a

*SD* standard deviation, *n/a* not applicable, as model only yielded two distributions

## Appendix 2

**Table 5:** Detailed cluster descriptions for those with alcohol dependence in both Wave 1 and Wave 2

	*N*	Age—mean (SD)	Alcohol consumption (g)—mean (SD)
Females			
Cluster 1			
Utilized treatment*	0	NA	NA
Did not utilize treatment	4	20.98 (5.84)	0.47 (0.25)
Cluster 2			
Utilized treatment*	21	37.78 (12.88)	31.94 (41.87)
Did not utilize treatment	105	34.38 (10.92)	24.42 (24.09)
Males			
Cluster 1			
Utilized treatment*	4	30.0 (0.0)	0.69 (0.0)
Did not utilize treatment	1	39.0 (0.0)	1.16 (0.0)
Cluster 2			
Utilized treatment*	28	33.52 (10.78)	17.16 (7.71)
Did not utilize treatment	102	30.71 (11.13)	17.07 (7.45)
Cluster 3			
Utilized treatment*	72	34.94 (11.09)	98.61 (58.16)
Did not utilize treatment	128	31.87 (12.39)	77.22 (42.61)

*g* grams, *SD* standard deviation

*Utilized treatment in the past 12 months at Wave 1 and/or between Wave 1 and Wave 2

## Appendix 3

The algorithm for calculating the average daily volume of ethanol intake (https://pubs.niaaa.nih.gov/publications/NESARC_DRM2/NESARC2DRM.pdf):

“First, all the reported frequencies of drinking were converted to number of drinking days per year, using the midpoints of the categorical response options—e.g., 3 to 4 times a week = 3.5 x 52 = 182. (For respondents who did not drink the type of beverage in question, the frequency was set to zero.)

For respondents whose largest quantity of drinks was 5 or fewer, average daily volume of ethanol intake had two components:

1. the usual quantity times the frequency of drinking that quantity: QU x FU, where FU = the overall frequency of drinking minus the frequency of drinking the largest quantity; and
2. the largest quantity times the frequency of drinking the largest quantity: QL x FL.

The sum of these two products, representing the total number of drinks consumed per year, was then multiplied by the ethanol content of the drink in ounces, derived by multiplying the size of drink in ounces times the ethanol content by volume. The resulting annual volume of ethanol intake was divided by 365 to yield average daily ethanol intake of the beverage in question. These volumes were then summed across beverages to yield the overall average daily volume of ethanol intake.

For respondents whose largest quantity of drinks was 6 or more, average daily volume had three components:

1. the usual quantity times the frequency of drinking that quantity: QU x FU, where FU = the overall frequency minus the frequency of drinking 5 or more drinks;
2. an intermediate component, Q5 x F5, where F5 = the frequency of drinking 5 or more drinks minus the frequency of drinking the largest quantity and Q5 = exp((log(max(5, QU)) + log( QL - 1)) /2)—that is, the geometric mean of the band of quantities between 5 and the largest number of drinks minus 1; and
3. the largest quantity times the frequency of drinking the largest quantity: QL x FL.

Again, this sum of products was multiplied by the ethanol content per drink and divided by 365 to yield average daily ethanol intake of the beverage in question, and volumes were summed across beverages to yield the overall average daily volume of ethanol intake.”

## Abbreviations

AUDs: Alcohol use disorders
AUDADIS-IV: Alcohol Use Disorders and Associated Disabilities Interview Schedule-IV
DSM-IV: Diagnostic and Statistical Manual of Mental Disorders, fourth edition
DSM-5: Diagnostic and Statistical Manual of Mental Disorders, 5th edition
ICD-11: International Classification of Diseases, 11th revision
NESARC: National Epidemiologic Survey on Alcohol and Related Conditions
GMMs: Gaussian Mixture Models

## References

[1] Shield K, Manthey J, Rylett M, Probst C, Wettlaufer A, Parry CD (2020). National, regional, and global burdens of disease from 2000 to 2016 attributable to alcohol use: a comparative risk assessment study. Lancet Public Health.

[2] Rehm J, Shield K, Gmel G, Rehm M, Frick U (2013). Modeling the impact of alcohol dependence on mortality burden and the effect of available treatment interventions in the European Union. Eur Neuropsychopharmacol.

[3] Rehm J, Gmel GE, Gmel G, Hasan OSM, Imtiaz S, Popova S, Probst C, Roerecke M, Room R, Samokhvalov AV, Shield KD, Shuper PA (2017). The relationship between different dimensions of alcohol use and the burden of disease-an update. Addiction.

[4] Heilig M, MacKillop J, Martinez D, Rehm J, Leggio L, Vanderschuren LJ. Addiction as a brain disease revised: why it still matters, and the need for consilience. Neuropsychopharmacology. 2021;22:1–9. 10.1038/s41386-020-00950-y PMC8357831 33619327

[5] World Health Organization (2018). ICD-11 for Mortality and Morbidity Statistics.

[6] American Psychiatric Association (2013). Diagnostic and statistical manual of mental disorders.

[7] Carvalho AF, Heilig M, Perez A, Probst C, Rehm J (2019). Alcohol use disorders. Lancet.

[8] Rehm J, Heilig M, Gual A (2019). ICD-11 for alcohol use disorders: not a convincing answer to the challenges. Alcohol Clin Exp Res.

[9] Martin CS, Langenbucher JW, Chung T, Sher KJ (2014). Truth or consequences in the diagnosis of substance use disorders. Addiction.

[10] Rehm J, Marmet S, Anderson P, Gual A, Kraus L, Nutt DJ, Room R, Samokhvalov AV, Scafato E, Trapencieris M, Wiers RW, Gmel G (2013). Defining substance use disorders: do we really need more than heavy use?. Alcohol Alcohol.

[11] Rehm J, Anderson P, Gual A, Kraus L, Marmet S, Nutt D (2014). The tangible common denominator of substance use disorders: a reply to commentaries to Rehm et al.(2013). Alcohol Alcohol.

[12] Nutt DJ, Rehm J (2014). Doing it by numbers: a simple approach to reducing the harms of alcohol. J Psychopharmacol.

[13] Singh GM, Danaei G, Farzadfar F, Stevens GA, Woodward M, Wormser D, Kaptoge S, Whitlock G, Qiao Q, Lewington S, di Angelantonio E, vander Hoorn S, Lawes CMM, Ali MK, Mozaffarian D, Ezzati M, Global Burden of Metabolic Risk Factors of Chronic Diseases Collaborating Group; Asia-Pacific Cohort Studies Collaboration (APCSC), Diabetes Epidemiology: Collaborative analysis of Diagnostic criteria in Europe (DECODE), Emerging Risk Factor Collaboration (ERFC), Prospective Studies Collaboration (PSC) (2013). The age-specific quantitative effects of metabolic risk factors on cardiovascular diseases and diabetes: a pooled analysis. PLoS One.

[14] Unger T, Borghi C, Charchar F, Khan NA, Poulter NR, Prabhakaran D (2020). 2020 International Society of Hypertension global hypertension practice guidelines. Hypertension.

[15] Ledermann S (1956). Alcool, Alcoolisme, Alcoolisation.

[16] Skog OJ (1985). The collectivity of drinking cultures: a theory of the distribution of alcohol consumption. Br J Addict.

[17] Yu G, Sapiro G, Mallat S (2011). Solving inverse problems with piecewise linear estimators: from Gaussian mixture models to structured sparsity. IEEE Trans Image Process.

[18] Bishop CM. Pattern recognition and machine learning. New York: Springer-Verlag; 2006.

[19] Becker JB, Koob GF (2016). Sex differences in animal models: focus on addiction. Pharmacol Rev.

[20] Grant BF, Chou SP, Saha TD, Pickering RP, Kerridge BT, Ruan WJ, Huang B, Jung J, Zhang H, Fan A, Hasin DS (2017). Prevalence of 12-month alcohol use, high-risk drinking, and DSM-IV alcohol use disorder in the United States, 2001-2002 to 2012-2013: results from the National Epidemiologic Survey on Alcohol and Related Conditions. JAMA Psychiatry.

[21] Ilgen MA, Price AM, Burnett-Zeigler I, Perron B, Islam K, Bohnert AS (2011). Longitudinal predictors of addictions treatment utilization in treatment-naïve adults with alcohol use disorders. Drug Alcohol Depend.

[22] Imtiaz S, Loheswaran G, Le Foll B, Rehm J (2018). Longitudinal alcohol consumption patterns and health-related quality of life: results from the National Epidemiologic Survey on Alcohol and Related Conditions. Drug Alcohol Rev.

[23] Grant BF, Dawson DA, Stinson FS, Chou PS, Kay W, Pickering R (2003). The Alcohol Use Disorder and Associated Disabilities Interview Schedule-IV (AUDADIS-IV): reliability of alcohol consumption, tobacco use, family history of depression and psychiatric diagnostic modules in a general population sample. Drug Alcohol Depend.

[24] Huang B, Grant BF, Dawson DA, Stinson FS, Chou SP, Saha TD, Goldstein RB, Smith SM, Ruan WJ, Pickering RP (2006). Race-ethnicity and the prevalence and co-occurrence of Diagnostic and Statistical Manual of Mental Disorders, alcohol and drug use disorders and Axis I and II disorders: United States, 2001 to 2002. Compr Psychiatry.

[25] Edition F. Diagnostic and statistical manual of mental disorders. Am Psychiatric Assoc. 2013;21.

[26] Hasin DS, Stinson FS, Ogburn E, Grant BF (2007). Prevalence, correlates, disability, and comorbidity of DSM-IV alcohol abuse and dependence in the United States: results from the National Epidemiologic Survey on Alcohol and Related Conditions. Arch Gen Psychiatry.

[27] Kehoe T, Gmel G, Shield KD, Gmel G, Rehm J (2012). Determining the best population-level alcohol consumption model and its impact on estimates of alcohol-attributable harms. Popul Health Metrics.

[28] Rehm J, Kehoe T, Gmel G, Stinson F, Grant B, Gmel G (2010). Statistical modeling of volume of alcohol exposure for epidemiological studies of population health: the US example. Popul Health Metrics.

[29] Charrad M, Ghazzali N, Boiteau V, Niknafs A. Determining the best number of clusters in a data set. J Stat Softw. 2014.

[30] Hartigan JA, Wong MA (1979). Algorithm AS 136: A k-means clustering algorithm. J R Stat Soc: Ser C: Appl Stat.

[31] Agency EM (2010). Guideline on the development of medicinal products for the treatment of alcohol dependence, EMEA/CHMP/EWP/20097/2008.

[32] Midanik LT (1988). Validity of self-reported alcohol use: a literature review and assessment. Br J Addict.

[33] Rehm J, Klotsche J, Patra J (2007). Comparative quantification of alcohol exposure as risk factor for global burden of disease. Int J Methods Psychiatr Res.

[34] Rehm J, Kilian C, Rovira P, Shield KD, Manthey J. The elusiveness of representativeness in general population surveys for alcohol. Drug Alcohol Rev. 2021;40(2):161–5. 10.1111/dar.13148 32830351

[35] Shield KD, Rehm J (2012). Difficulties with telephone-based surveys on alcohol consumption in high-income countries: the Canadian example. Int J Methods Psychiatr Res.

[36] Roesner S, Hackl‐Herrwerth A, Leucht S, Lehert P, Vecchi S, Soyka M. Acamprosate for alcohol dependence. Cochrane Database Syst Rev. 2010(9). 10.1002/14651858.CD004332.pub2 PMC12147086 20824837

[37] Roesner S, Hackl‐Herrwerth A, Leucht S, Vecchi S, Srisurapanont M, Soyka M. Opioid antagonists for alcohol dependence. Cochrane Database Syst Rev. 2010(12). 10.1002/14651858.CD001867.pub3 PMC13590218 21154349

[38] Rehm J, Manthey J, Struzzo P, Gual A, Wojnar M (2015). Who receives treatment for alcohol use disorders in the European Union? A cross-sectional representative study in primary and specialized health care. Eur Psychiatry.

[39] Rehm J, Allamani A, Aubin H-J, Della Vedova R, Elekes Z, Frick U (2015). People with alcohol use disorders in specialized care in eight different European countries. Alcohol Alcohol.

[40] Fan J, May SJ, Zhou Y, Barrett-Connor E (2005). Bimodality of 2-h plasma glucose distributions in whites: the Rancho Bernardo study. Diabetes Care.

[41] Culverhouse R, Bucholz KK, Crowe RR, Hesselbrock V, Nurnberger JI, Porjesz B, Schuckit MA, Reich T, Bierut LJ (2005). Long-term stability of alcohol and other substance dependence diagnoses and habitual smoking: an evaluation after 5 years. Arch Gen Psychiatry.

[42] Rehm J, Guiraud J, Poulnais R, Shield KD (2018). Alcohol dependence and very high risk level of alcohol consumption: a life-threatening and debilitating disease. Addict Biol.

[43] Meiklejohn J, Connor J, Kypri K (2012). The effect of low survey response rates on estimates of alcohol consumption in a general population survey. PLoS One.

[44] Rose G. The population strategy of prevention. Strategy Prev Med. 1992:95–106.

